# Morphological, anatomical, and mechanical variation in cactus spines across species and stem developmental positions

**DOI:** 10.1093/aobpla/plag050

**Published:** 2026-10-05

**Authors:** Roman Gebauer, Radomír Řepka, Radim Matula, Daniel Volařík, Jan Tippner, Barbora Vojáčková

**Affiliations:** Department of Forest Botany, Dendrology and Geobiocoenology, Faculty of Forestry and Wood Technology, Mendel University in Brno, Zemědělská 3, Brno 61300, Czech Republic; Department of Forest Botany, Dendrology and Geobiocoenology, Faculty of Forestry and Wood Technology, Mendel University in Brno, Zemědělská 3, Brno 61300, Czech Republic; Department of Forest Ecology, Faculty of Forestry and Wood Sciences, Czech University of Life Sciences Prague, Kamýcká 129, Prague 165 00, Czech Republic; Department of Forest Botany, Dendrology and Geobiocoenology, Faculty of Forestry and Wood Technology, Mendel University in Brno, Zemědělská 3, Brno 61300, Czech Republic; Department of Wood Science and Wood Technology, Faculty of Forestry and Wood Technology, Mendel University in Brno, Zemědělská 3, Brno 61300, Czech Republic; Department of Wood Science and Wood Technology, Faculty of Forestry and Wood Technology, Mendel University in Brno, Zemědělská 3, Brno 61300, Czech Republic

**Keywords:** anatomy, Cactaceae, fibre, modulus of elasticity, modulus of rupture, spine development, spine structure, toughness

## Abstract

Cactus spines are highly variable structures that perform multiple ecological functions and have considerable taxonomic importance. However, their anatomical and mechanical properties remain poorly understood. A better understanding of spine structure may provide insights into the ecological function. In this study, we compared morphological, anatomical, and mechanical spine traits in three columnar cacti from Argentina (*Cereus hankeanus*, *Stetsonia coryne,* and *Trichocereus terscheckii*) and evaluated how these traits vary along a developmental gradient extending from the apical meristem. Spines were sampled from a single rib of 10 individuals per species. We measured spine length, fibre dimensions, cell wall thickness, and performed mechanical tests on oven-dried spines to calculate modulus of elasticity, modulus of rupture, and toughness. Significant differences in spine traits were detected among species. *Stetsonia* had the longest spines and exhibited the highest toughness, whereas *Trichocereus* showed lower mechanical resistance despite having thicker cell walls. Anatomical variation was also evident, with *Stetsonia* having smaller and more elliptical fibres than *Trichocereus*. In addition to interspecific differences, spine length in *Stetsonia* and *Cereus* increased with distance from the apex, indicating developmental changes along the stem. Among mechanical traits, only toughness varied along the stem, decreasing with increasing distance from the apex in all species, while anatomical traits remained unchanged. Together, these findings reveal substantial interspecific, intraspecific, and positional variation in cactus spine traits, highlighting the influence of developmental position along the stem and spine ageing, as well as species-specific differences in spine structure and mechanical performance.

## Teaser

This study reveals that cactus spines are far more complex and variable than their outward appearance suggests. By examining the morphology, anatomy, and mechanical properties of spines in three columnar cactus species, substantial differences among species were identified, as well as variation within individual plants. Spine traits were influenced not only by species identity but also by developmental position and ageing along the stem. Importantly, no single trait fully captured spine diversity, highlighting the need to consider structural and mechanical characteristics together. These findings improve our understanding of cactus spine development and function, with important implications for ecology, taxonomy, and the interpretation of spine diversity in cacti.

## Introduction

Spines are probably the most distinctive features of almost all species within the family of Cactaceae. A number of beneficial functions have been ascribed to spines, including direction of intercepted water towards roots (Benson 1982), protection from herbivory (McAuliffe 1984, Norman and Martin 1986), insulation of sensitive meristems from freezing temperatures (Nobel 1988, Loik and Nobel 1993), and shading photosynthetic cortex from intense radiation and UV exposure (Loik 2008, de la Rosa-Manzano *et al*. 2016, Aliscioni *et al*. 2021). The ecological relevance of spines may extend beyond their mere presence or absence, as variation in their morphology and mechanical properties may alter their functional effectiveness. This notion is supported by studies showing that the size and density of plant defensive structures can strongly influence plant–herbivore interactions and may reflect adaptation to local herbivory pressure. For example, *Acacia sieberiana* individuals exposed to high browsing pressure invested in longer and more numerous thorns, which reduced damage caused by large mammalian herbivores (Babah Daouda *et al*. 2025). Although variation in spine morphology and mechanical properties appears to have important ecological consequences, understanding of their contribution to ecological function remains limited. This knowledge gap is partly caused by the poor characterization of spine trait variation within species and individual plants, limiting our ability to link spine structure and mechanical properties to their functional roles. Understanding the magnitude and sources of this variation is therefore needed for interpreting the ecological and taxonomic significance of spine traits.

Spines vary considerably across species (Mauseth 2006), and this large morphological variation has led to the widespread use of traits, such as distance between areoles, number of spines per areole, spine length, and number of ribs for classification taxa within Cactaceae (e.g. Schmalzel *et al*. 2004, Hunt *et al*. 2006). Although spine traits have been shown to be at least partly heritable (Mihalte and Sestras 2012), substantial variation within species has been documented across a range of cactus taxa (English *et al*. 2007, 2010, Drezner 2017, Mauseth 2017, Hultine *et al*. 2019). This suggests that spine traits are influenced by multiple sources of variation, including genetic and environmental factors. Spine length is among the most frequently used taxonomic characters because it is easy to measure and often differs among taxa. However, previous studies have documented substantial intraspecific variation in spine length (Rundel 1977, Gebauer *et al*. 2016), which limit its reliability as a standalone diagnostic trait. Existing evidence suggests that spine traits are highly responsive to environmental conditions. For example, stems of *Opuntia pusilla* (Haw.) Haw. grown in the full sunlight presented dense spine coverage, while shaded stems grew with few or no spines (Majure 2007). Similarly, populations of *Carnegiea gigantea* exposed to more extreme thermal and xeric conditions develop longer and denser spines (Drezner 2017). These observations indicate that variation in spine traits is likely to be functionally and ecologically significant.

Spines develop from areoles on the photosynthetic cactus stem, and both areoles and spines are displaced away from the stem apex as new areoles are produced during growth (Gibson and Nobel 1986, Mauseth 2006). Although spines formation is completed within a few weeks (approximately 30–60 days), spines persist along the stem for decades without further elongation (English *et al*. 2010, Miravel-Gabriel *et al*. 2024). Unlike woody plants, however, columnar cacti do not form visible annual growth rings, which makes the assessment of absolute spine age difficult based solely on the areole order (Delgado-Fernández *et al*. 2016, Hultine *et al*. 2018, 2019). However, spines retain biochemical and isotopic signatures that record physiological and environmental conditions prevailing during their formation, allowing the reconstruction of chronological and environmental histories along cactus stems using stable carbon isotope (δ^13^C) and radiocarbon (^14^C) dating (English *et al*. 2010, 2021, Hultine *et al*. 2018). Because spines formed at different positions along the stem differ in developmental age and may reflect environmental conditions prevailing during their formation, variation among them represents an important source of within-plant trait variability. This pattern is consistent with growing evidence that substantial variation can occur among organs within a single plant, potentially influencing structure–function relationships (Herrera 2009, Westerband *et al*. 2021). Indeed, studies of leaf traits have shown that variation among organs of a single individual can equal or even exceed variation among individuals and populations (Herrera *et al*. 2015, Sánchez-Bermejo *et al*. 2023). Although comparable studies are scarce for cactus spines, existing evidence suggests that spine traits are highly variable and responsive to environmental conditions. Consequently, spine position along the stem should be considered when interpreting morphological, anatomical, and mechanical traits. This substantial variability also implies that appropriate sampling intensity and sampling position are critical when spine traits are used for taxonomic or ecological analyses (Cubas *et al*. 2025).

Over decades, many studies have investigated the morphology of cacti spines, but only a few have focused on their anatomical and mechanical properties (e.g. Mosco 2009, Gindl-Altmutter and Keckes 2012, Huang and Guo 2013). While morphological traits describe the external form of spines, anatomical traits characterize their internal structure, and mechanical traits quantify their physical performance (e.g. stiffness, strength, or toughness). Among cacti, the anatomical structure of spines is generally simple, consisting of a core of longitudinally oriented fibres surrounded by a single-layered spine epidermis, whereas a multiple-layered epidermis occurs only rarely (Mosco 2009; Řepka and Gebauer 2012, Gebauer *et al*. 2016). Integrating these levels of organization is therefore important for improving our understanding of how spine structure is associated with mechanical performance. Several studies have assessed the mechanical properties of cactus spines across different species, yielding highly variable results (Malainine *et al*. 2003, Schlegel 2009, Gindl-Altmutter and Keckes 2012, Cooper *et al*. 2013, Huang and Guo 2013, Martinez *et al*. 2017, Kundanati et el. 2022). However, because studies have examined different mechanical traits, and employed different methodologies, reported values are often not directly comparable. As a result, it is difficult to generalize how mechanical traits vary among species or how they are related to anatomical traits, as studies rarely integrate multiple levels of trait variation within a consistent framework.

To address this gap, we compared morphological, anatomical, and mechanical spine traits in three columnar cactus species growing under natural conditions in Argentina. These species belong to a group of cacti that occurs across a wide range of arid and semi-arid environments in the Americas and play important ecological roles (Alcorn *et al*. 1961, Fleming *et al*. 1996). We aimed to quantify differences in morphological, anatomical, and mechanical spine traits among species and to investigate how these traits vary with distance from the stem apex. We hypothesized that (i) species would differ not only in morphological but also in anatomical, and mechanical traits and that (ii) morphological, anatomical, and mechanical spine traits would vary with position along the stem, reflecting within-plant variation associated with developmental position and spine age. By jointly analysing morphological, anatomical, and mechanical traits, we evaluate whether commonly used spine characteristics provide consistent information across organizational levels and improve the interpretation of spine variation in taxonomic, ecological, and functional contexts.

## Material and methods

### Plant material

For the study of morphological, anatomical, and mechanical spine traits of columnar cacti, three abundant Argentinean species were selected: (i) *Stetsonia coryne* (Salm-Dyck) Britton & Rose (Fig. 1a), monotypic endemic species of the ecoregion Chaco, abundant in the north-western Argentina, but also in the western part of Paraguay, and south-eastern Bolivia. It grows on dry slopes of rock decay, shallow drying soil, but also on saline edges. Individuals are 5–8 m tall and have a shrubby or treelet growth form. Branches have 8–9 ribs, i.e. a longitudinal stem ridges bearing a sequence of areoles, which are densely covered with spines; (ii) *Cereus hankeanus* F. A. C.Weber ex K. Schum. (syn.: *C. forbesii* Otto ex C. Foerst.) (Figure 1b) has similar geographic distribution and ecological niche to the former species; it is shrubby or treelike sparsely branched species 2–4(−7) m high, branches with 4–7 ribs; (iii) *Trichocereus terscheckii* (J. Parm. ex Pfeiff.) Britton & Rose (Fig. 1c), which has relatively wide distribution range in Argentina (Catamarca, Salta, Jujuy, La Rioja, San Juan, and Tucumán provinces) and Bolivia (Tarija). It grows on various soil types in grasslands, shrublands, semiarid intermediate Chaco non-forest habitats, and mountain slopes ranging from 800 to 2000 m a.s.l. They are robust, 5–8(−12) m high, candlestick-like habitus, little branched columnar cacti, branches with 12–16 ribs (Lodé 2015, LLIFLE Encyclopedia of Cacti n.d.).

**Figure 1:**
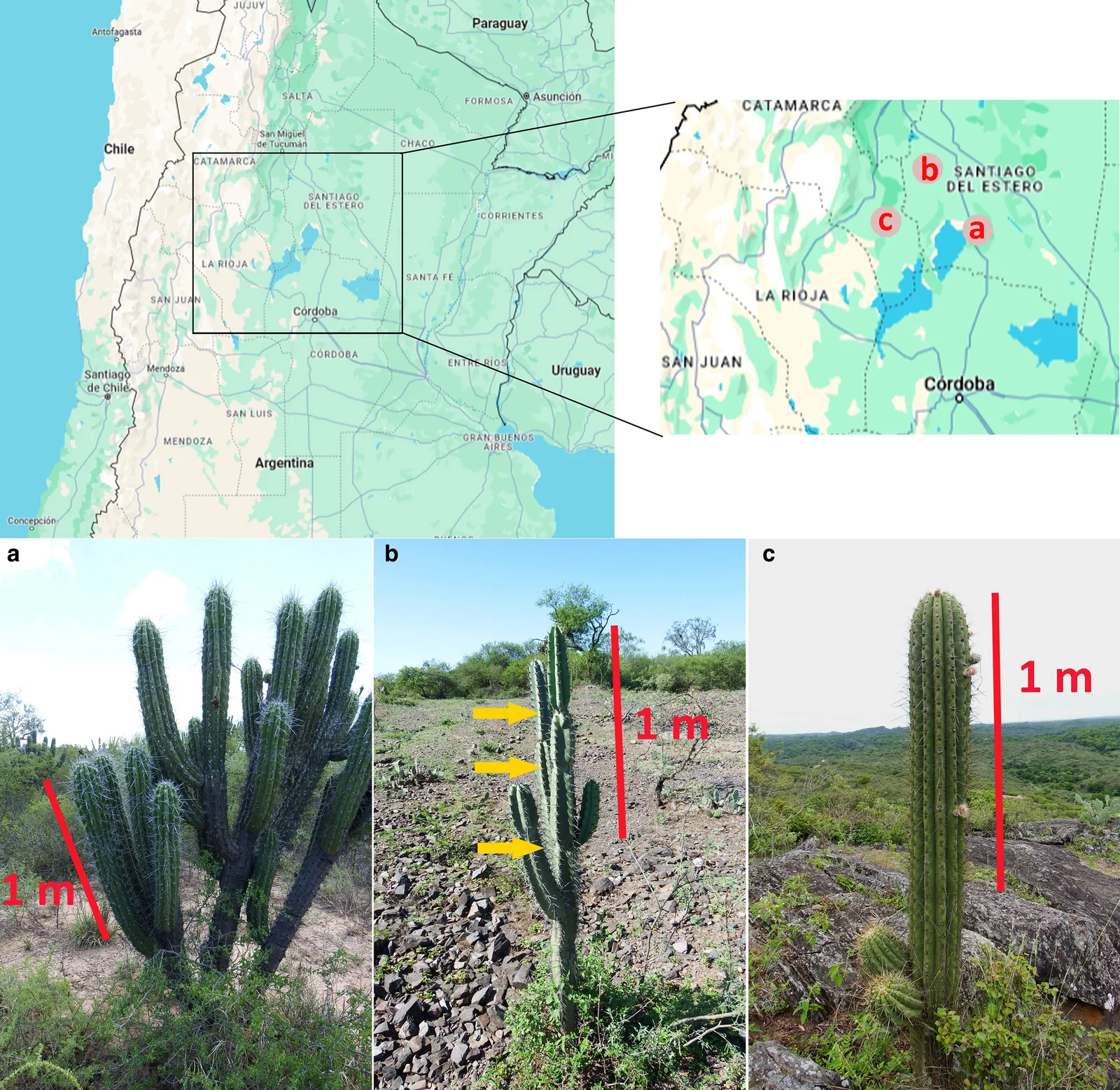
Selected columnar cacti growing in Argentina. (a) *Stetsonia coryne* (Salm-Dyck) Britton & Rose; (b) *Cereus hankeanus* F. A. C.Weber ex K. Schum.; (c) *Trichocereus terscheckii* (J. Parm. ex Pfeiff.) Britton & Rose. The sampling length is indicated by a red line. The 3-year annual stem increment of *Cereus hankeanus* is clearly visible and marked by yellow arrows.

All three species belong to the family Cactaceae (tribe *Cereeae*), indicating a shared evolutionary origin. A recent phylogenetic study places *Cereus hankeanus* and *Stetsonia coryne* within the subtribe *Cereinae*, whereas species *Trichocereus terscheckii* is placed within the subtribe *Trichocereinae* (de Vos *et al*. 2025). In the following text, only the generic names of the species are used: *Stetsonia, Cereus,* and *Trichocereus*.

### Study site and sampling

*Stetsonia* individuals were collected in Chaco árido near Lucio V. Mansilla (Córdoba Province), close to Salinas Grandes, at 230 m a.s.l. (S 29°48.44032’, W 64°41.91285’) (Fig. 1a). The *Cereus* population was studied on limestone hills near Villa Guasayan (Santiago del Estero Province) at 520 m a.s.l. (S 27°51.49923’, W 64°50.24412’) (Fig. 1b). The *Trichocereus* population was measured on rocky hillsides near Oyolas (Catamarca Province), at 910 m a.s.l. (S 28°27.72778’, W 65°23.67242’) (Fig. 1c). All sampling was done in November and December 2016. The sum of precipitation, and the mean daily maximum and minimum temperatures for all three localities from 2012 to 2016 were extracted from the highest-resolution (30 arc-seconds) WorldClim version 2.1 database (Fick and Hijmans 2017) (Fig. 2). The data show that precipitation peaked in 2014 and 2015, whereas temperatures generally reached their lowest values in 2015.

**Figure 2:**
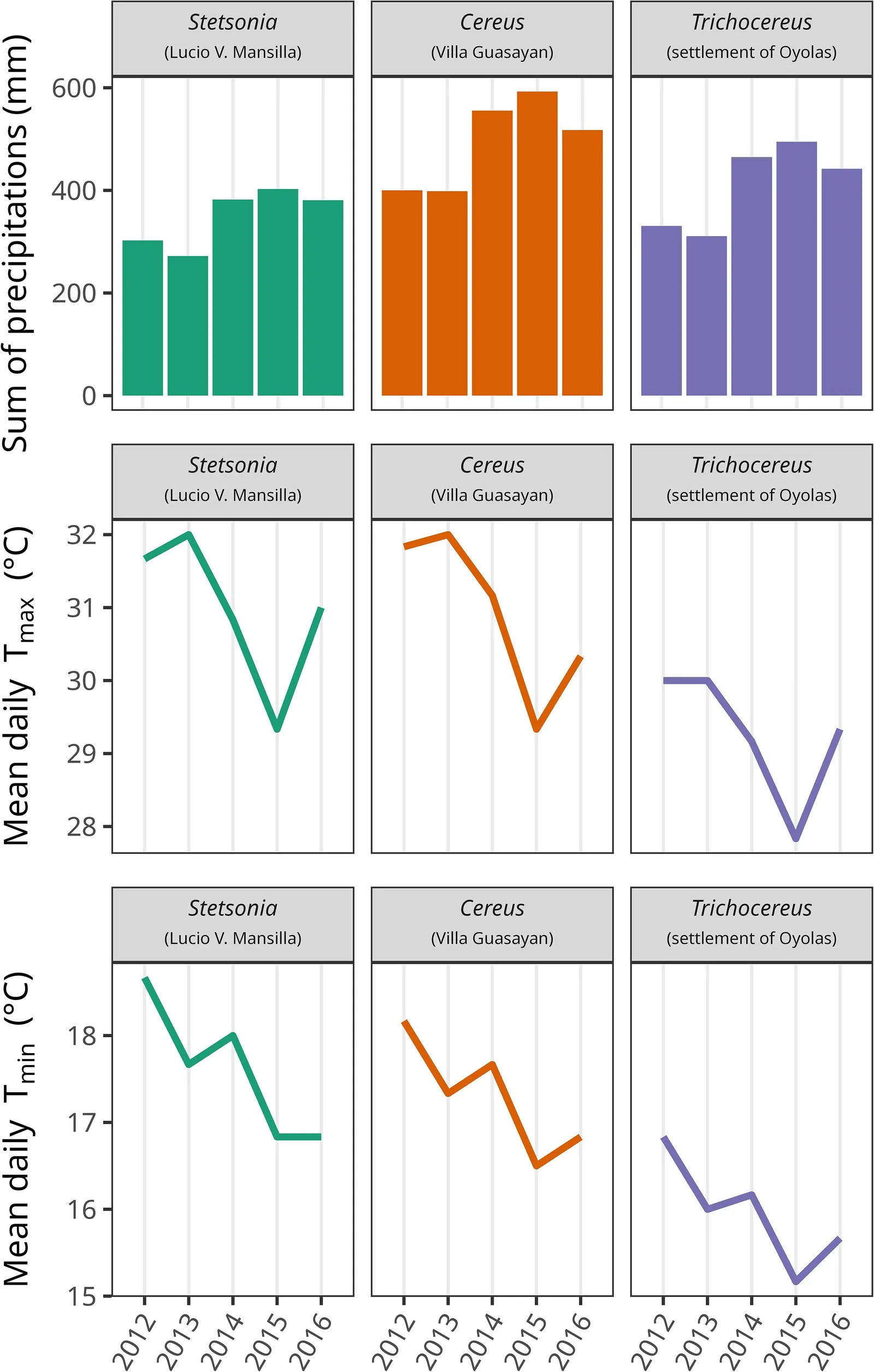
Sum of precipitation, and mean daily maximum and minimum temperatures in vegetation season (from September to February) for all three localities during the year of sampling and four preceding years extracted from WordClim dataset.

Ten healthy, solitary individuals with accessible stem apices were selected on each study site for each species. Spines were collected along a single cactus rib oriented to north (starting from the stem apex). The length of the cactus rib ranged from 1.0 to 1.4 m, 0.8 to 1.3 m, and 0.7 to 1.1 m for *Stetsonia*, *Trichocereus,* and *Cereus*, respectively. In *Cereus*, the annual increment of the stem was clearly visible (Fig. 1b), and spines from the last 3 years were collected. For all three species, the length of the longest spine in each areole was measured on the cactus rib (*S_l_*). The longest spines for anatomical and mechanical analysis were collected at regular distances from the stem apex. The exact areole order from stem apex, from which spines were collected, was recorded and used in statistical analyses. Six to eight spines per individual for *Stetsonia* (*n* = 67), seven spines per individual for *Trichocereus* (*n* = 70) and three spines per annual growth (from upper, middle, and basal parts) from the last 3 years for *Cereus* (*n* = 90) were collected. Because anatomical and mechanical analyses require different sample preparation, only subsets of the collected spines were used for each analysis. The subsampling strategy differed among species to maintain representative coverage along the stem axis and independence between anatomical and mechanical measurements.

### Anatomical analysis

Anatomical analyses were conducted for *Stetsonia* and *Trichocereus* using a subset of the collected spines. To capture variation associated with increasing distance from the apical meristem, spines were sampled from different positions along the stem axis. This included all spines from six *Stetsonia* individuals (*n* = 41) and spines from the upper, middle, and basal parts of the rib in each *Trichocereus* individual (*n* = 30). A 5% solution of hydrochloric acid was used to soften spines before sectioning. The spine length (*S_l_*) was measured before making cross-sections. Handmade cross-sections were taken from the spine base and were examined under a bright field microscope (Olympus BX51, Olympus Optical Co., Tokyo, Japan) at magnifications up to 400× and were photographed using a digital camera (Olympus E-330, Olympus Czech Group, Czech Republic) connected to a computer with the Quick Photomicro 2.3 software (Promicra, Czech Republic) (Supplementary Figure S1). Since the fibres taper at the ends, only the 10 thickest fibres (selected from 15 to 20 measured fibres and visually identified as the thickest) per spine were selected on each cross-section. Then, fibre maximum and minimum diameters (*d_max_* and *d_min_*), and cell wall thickness (*CW_th_*) were measured using the ImageJ 1.49 software (UTHSCSA, USA) (Supplementary Figure S2). Fibre roundness (*F_r_*) was defined as the *d_max_/d_min_* ratio, with a ratio of 1 denoting a perfectly round cross-section and larger ratios indicating a more ellipsoidal shape of cross-section.

### Mechanical analysis

For mechanical analysis, a separate subset of spines was used to avoid any influence of anatomical preparation on mechanical properties. For *Stetsonia*, all spines from four individuals were used (*n* = 26). For *Trichocereus*, four spines were used from the upper, middle, and basal parts of the rib of each of 10 individuals (*n* = 40). In this species, spines were selected along the rib in a systematic sequence to ensure comparable representation of positions along the stem for both anatomical and mechanical datasets. For *Cereus*, a total of six spines (three from the first-year increment and three from the third-year increment) were used from each of 10 individuals (*n* = 60). The samples were completely dried in a kiln SANYO MOV-212P (Sanyo Electric Co., Ltd., Moriguchi, Japan) for 2 days at 103°C and weighed to achieve a steady dried condition. The mechanical three-point bending tests were performed to obtain a force-displacement curve during loading up to the crack of the spines. The spines were destructively tested within 10 minutes after drying to avoid the influence of temperature and moisture content increase. Due to the difficulties of measuring the mechanical properties of spines, the measurement of mechanical traits could not be carried out for 14 spines of *Cereus* and 7 spines of *Trichocereus*. In total, 105 oven-dry spines were mechanically tested by a three-point bending test.

Mechanical testing machine Tinius Olsen 10 ST (Tinius Olsen Testing Machine Company, USA) with sensitive force head (max. loading capacity 500 N) was used (Supplementary Figure S3). Test procedure and data acquisition were controlled by Horizon Software v10.0.8.0 T (Tinius Olsen Testing Machine Company, USA). The tests were position-controlled (velocity of loading 4 mm.min^−1^), with a cylindrical supporting and loading bars (10 mm diameter) with a support span of 26 mm. Spines were not fixed at the supports.

The middle diameter of spines, *d* (mm), the ultimate (break) force, *F_max_* (N; the maximum force a spine can withstand before breaking), and the deflection, *u_max_* at *F_max_* (mm; the amount of bending at the point of failure) were measured. The modulus of elasticity (*MOE*; a measure of stiffness, describing resistance to bending under load) and bending strength, expressed of the modulus of rupture (*MOR*; the maximum stress sustained before failure), were derived similarly to Milch et al. (2016) with the consideration of circular cross–section (similarly to Schlegel (2009)) as presented in the following equations 1 and 2:


(1) $$MOE=\frac{4l^{3}(F_{40}-F_{10})}{3\pi d^{4}(u_{40}-u_{10})}$$

(2) $$MOR=\frac{8lF_{max}}{\pi d^{3}}$$

where *l* is the support span length, *d* is the diameter in the middle of the spine, *F_10,40_* is the force measured at 10% and 40% level of maximum force (*F_max_*), and *u_10,40_* is the displacement at the level of *F_10,40_*.

Static work *W_S_* (N.mm; the energy absorbed by a spine during bending until failure, reflecting material toughness) was calculated as an integral below the force-displacement curve by trapezoidal numerical integration (MATLAB Version: 9.14.0.2286388, R2023 a, Update 3, The MathWorks, Inc., Natick, MA, USA). Static work was divided by the circle cross-section area to obtain toughness (*K_S_*) of the spines (J.mm^−2^).

### Statistical analysis

We used (generalized) linear mixed effect models (GLMM) for statistical analysis due to the hierarchical nature of our data where fibres were nested within spines and spines where nested within plants. Then, we estimated statistical models for anatomical traits with a random effect of spine nested in plant (varying intercepts; varying slopes of areole order at plants level). The same approach was used for mechanical traits and spine length, except that the spine level was omitted as these traits were only measured at the spine level (not at the fibres level). One model was fitted for each trait, incorporating previously described random effects and with fixed effects of species, areole order, and their interaction. For almost all models, we used normal probability distribution family to model the spread of data. The exception was *F*_r_, for which the data where left-skewed and therefore a lognormal distribution was used.

We used a Bayesian approach for all models. The model parameters were estimated using the Markov chain Monte Carlo (MCMC) method as implemented in Stan (Stan Development Team 2025), via the brms package version 2.23.0 (Bürkner 2017, 2018) in the R software environment version 4.5.3 (R Core Team 2025). Graphics were produced using the ggplot2 package (Wickham 2016). The choice between Bayesian and frequentist approaches is often based on personal preferences. In this study, however, selecting the Bayesian approach combined with the MCMC method to estimate the parameters enabled us to obtain reliable parameter estimates, since the number of sampled plant individuals was rather low for all traits except spine length. We used weakly informative default priors from the brms package for most parameters. However, for main effect-size parameters (i.e. differences between species, the slope of areole order for *Stetsonia*, and differences in slopes for other species), we specified weakly regularizing priors instead. These priors were normally distributed and centred at zero, with standard deviations defined as follows: twice the standard deviation of the response variable for differences between species; and the range of the response divided by the maximum areole order for slopes and slope differences (check the R script in the supplementary data for more details). We performed a prior sensitivity analysis for all models using the priorsense package (version 1.2; Kallioinen *et al*. 2023). This procedure detects parameters for which prior distribution may conflict with the data, i.e. where prior distribution has a strong influence on the posterior distribution. We applied this check to all models and parameters, and found no evidence of prior–data conflict. All models were fitted using four chains with 6000 iterations per chain, including 1000 warm-up iterations. The result tables summarize the posterior distributions of the key parameters: the mean value for *Stetsonia*; the differences for the other two species at the 5th, 15th, and 25th areole orders; and the slopes describing the effect of the areole order for all species. The 5th, 15th, and 25th areole orders were selected as representative reference positions corresponding to the apical, middle, and basal stem regions, respectively. The fifth areole was chosen as the uppermost reference position because spines at this stage are already fully developed. These summaries are presented as median values and 95% credible intervals (CI), along with the probability of direction (pd), i.e. the proportion of posterior distribution that is on the median’s sign (Makowski *et al*. 2019 for a detailed description). Whenever a term ‘discernible’ is used, it refers to a 0.975 threshold of pd. Furthermore, conditional and marginal *R*^2^ was calculated using the r2_bayes function from the performance package version 0.15.1 (Lüdecke *et al*. 2021).

We also refitted all models, using a standardized response variable (i.e. the mean value was subtracted, and was scaled by the standard deviation), and an areole order centred on the fifth areole. These models were fitted to obtain standardized standard deviations, particularly at the plant level, so that they could be compared across traits. Next, to obtain a general overview of the species effect, we fitted a simpler version of each model without the species effect. In the case of spine length, CW_th_, *F*_r_, and *K*_s_, we also fitted an additional model that included only the additive effect of species. We assessed the predictive accuracy of the model with and without the species effect by calculating the difference in expected log pointwise predictive density (ΔELPD) using the leave-one-out cross-validation method. ELPD was calculated without including random effects associated with individual identifiers (particularly plant ID), because these effects overlap with and may obscure the species effect. Further details of the data analyses can be found in the supplementary data, which includes raw data tables and R scripts for all the statistical analyses.

## Results

### Species comparison

In general, species was an important predictor of the studied traits (Table 1). Discernible differences were detected among at least some species pairs and for at least some areole orders (Supplementary Table S1). The effect of spines on toughness (*K*_s_) was weaker (Table 1); nevertheless, detailed analyses also revealed discernible differences in *K*_s_ (Supplementary Table S1).

**Table 1:** The overall effect of species on the studied traits and the variation in these traits at the plant and spine levels are presented.

	Trait	Overall species effect		Variation expressed by standard deviation						*R* ^2^ conditional	*R* ^2^ marginal
		ΔELPD	Standard error of ΔELPD	At Plant level (95% CI)—at Cacti branch apex		At Spine level (within plant) (95% CI)—at Cacti branch apex		Residual (95% CI)			
morholo-gical	spine length	284.1	39.0	14.150	(10.631, 19.374)	—	—	12.945	(12.332, 13.594)	0.608	0.323
anatomical	*d_max_*	221.1	41.1	0.818	(0.050, 1.991)	1.404	(1.040, 1.878)	1.260	(1.179, 1.349)	0.714	0.283
	*d_min_*	449.7	60.4	0.727	(0.038, 2.094)	1.332	(0.926, 1.774)	1.103	(1.033, 1.180)	0.751	0.340
	*F_r_* ^a^	30.9	11.2	0.029	(0.001, 0.109)	0.091	(0.069, 0.120)	0.116	(0.109, 0.125)	0.451	0.114
	*CW_th_*	200.3	37.3	0.620	(0.053, 1.279)	0.559	(0.363, 0.799)	0.539	(0.503, 0.577)	0.683	0.218
mechanical	*MOR*	22.8	8.7	52.98	(2.68, 154.62)	—	—	235.26	(201.76, 274.08)	0.408	0.292
	*MOE*	24.0	8.5	2.646	(0.116, 8.533)	—	—	13.993	(12.018, 16.376)	0.439	0.288
	*K_S_*	7.3	4.6	0.002	(0.001, 0.006)	—	—	0.009	(0.008, 0.011)	0.338	0.244

The species effect is quantified as the difference in the expected log pointwise predictive density (ΔELPD) for a new dataset between a model including species (either as an additive or synergic effect) and a model without species. A positive ΔELPD values indicate improved predictive accuracy of the model that includes species. For all studied traits, models including species showed higher predictive accuracy, indicating that species is an important predictor and that at least some species pairs differ in the studied traits. Detailed pairwise comparison for the three selected areole orders are provided in Supplementary Table S1. For *K*_s_, however, the standard error of ΔELPD is as large as the estimated difference itself, suggesting considerable uncertainty in the improvement. Trait variation at the cacti branch apex is described by the standard deviation at the plant and spine levels. The residuals (errors) standard deviation is also reported. In addition, conditional *R*^2^ (including both fixed and random effects) and marginal *R*^2^ (fixed effects only) are presented. Results are reported as point estimates (posterior medians) with 95% credible intervals (CIs). Estimates are derived from the same statistical models used in Supplementary Tables S1 and S2, with a separate model fitted for each trait.

^a^ *F_r_* was fitted with log-normal distribution, standard deviations are therefore on natural logarithm scale.

*Stetsonia* had the longest spines among the studied columnar cacti species (Fig. 3; Supplementary Table S1). Spine length exceeded that of *Cereus* by 20.7–24.1 mm and that of *Trichocereus* by 21.6–34.1 mm, depending on the areole position, with all pairwise comparisons receiving very strong posterior support (pd ≥ 0.999) (Supplementary Table S1). In contrast, differences between *Cereus* and *Trichocereus* were small (0.9–10.0 mm) and had weaker posterior support (pd = 0.562–0.934), indicating similar spine lengths in these two species (Supplementary Table S1). Anatomical analysis revealed consistent differences between *Stetsonia* and *Trichocereus* (Fig. 4; Supplementary Table S1). Compared with *Trichocereus*, *Stetsonia* had smaller maximum and minimum fibre diameters ((*d_max_* and *d_min_*), which were lower by 2.1–3.0 µm and 2.5–2.6 µm, respectively (pd ≥ 0.986) (Supplementary Table S1). In addition to having smaller fibre diameters, fibres of *Stetsonia* were generally more ellipsoid than those of *Trichocereus*, with differences in fibre ratio (*F_r_*) of 0.17 and 0.12 at the 5th and 15th areole orders, respectively (pd ≥ 0.993) (Fig. 4; Supplementary Table S1). The species also differed in cell wall thickness (*CW_th_*) by 0.83–0.88 µm at the 5th and 15th areole orders (pd ≥ 0.990) (Fig. 4; Supplementary Table S1).

**Figure 3:**
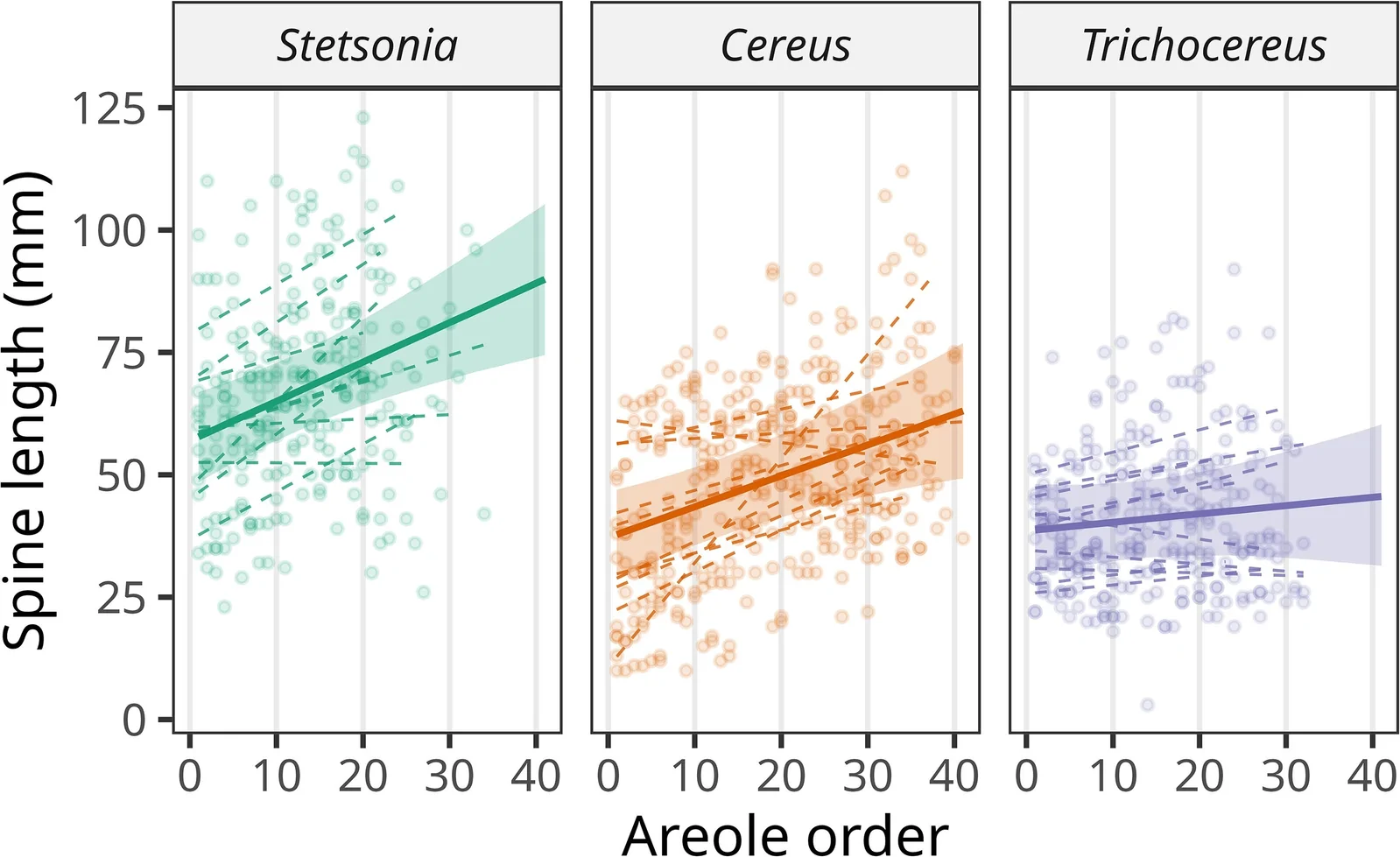
Spine length variation along a cactus rib for three columnar cacti species—*Stetsonia coryne*, *Cereus hankeanus* and *Trichocereus terscheckii*. The mean trends for plant individuals are shown by a dashed line, and the population mean trend for each species is shown by a solid line together with 95% credible interval represented by the shaded area. Points represent measured values. It shows that *S. coryne* has the longest spines, although spine length overlaps among all species, and increases along the rib in *S. coryne* and *C. hankeanus*.

**Figure 4:**
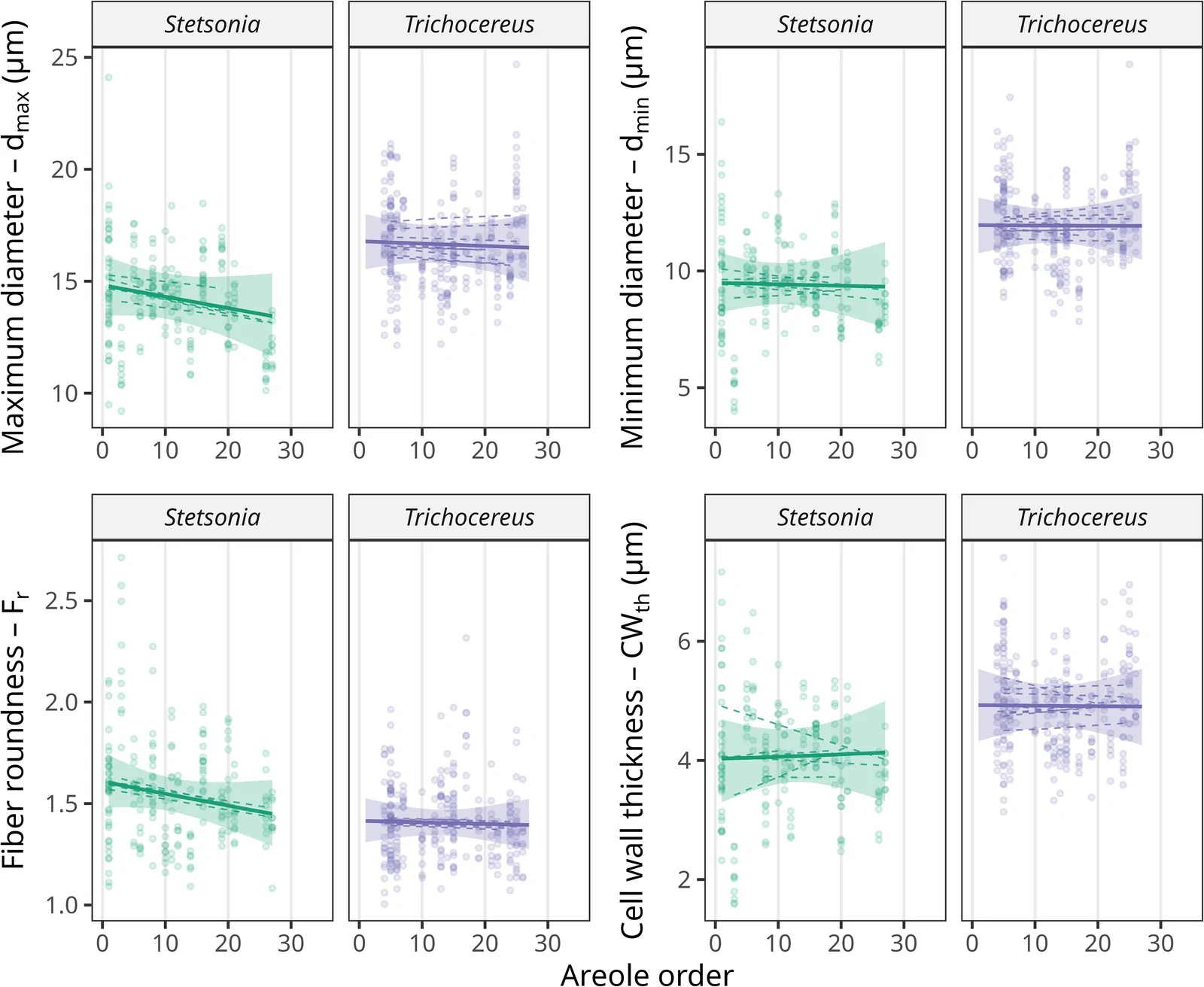
Spine anatomical traits variation along a cactus rib for two columnar cacti species—*Stetsonia coryne* and *Trichocereus terscheckii*. Fibre maximum diameter (*d_max_*), fibre minimum diameters (*d_min_*), cell wall thickness (*CW_th_*), and fibre roundness (*F_r_*) are shown. The mean trends for plant individuals are shown by a dashed line, and the population mean trend for each species is shown by a solid line together with 95% credible interval represented by the shaded area. Points represent measured values. Spine anatomy differs between species, but overlaps and the effect of areole order is very small, it is not statistically discernible.

Mechanical properties were similar in *Stetsonia* and *Cereus* across all tested areole orders (Fig. 5; Supplementary Table S1). Estimated differences in modulus of rupture, modulus of elasticity, and toughness were small (24–79 MPa, 0.5–3.1 GPa, and 0.002–0.007 J mm^−2^, respectively) and generally received limited posterior support (pd = 0.582–0.920) (Fig. 5; Supplementary Table S1). By contrast, *Trichocereus* spines exhibited lower mechanical traits than both *Stetsonia* and *Cereus* (Supplementary Table S1). Relative to *Stetsonia*, modulus of rupture was lower by 276–313 MPa (pd ≥ 0.985), and modulus of elasticity by 16.5–23.2 GPa (pd ≥ 0.995). Comparable differences were observed relative to *Cereus*, with modulus of rupture lower by 247–391 MPa (pd ≥ 0.997) and modulus of elasticity lower by 13.4–22.6 GPa (pd ≥ 0.995) (Supplementary Table S1). *Trichocereus* also exhibited lower toughness values than *Stetsonia* and *Cereus* at the 5th and 15th areole orders (Supplementary Table S1). Differences ranged from 0.007 to 0.011 J mm^−2^ relative to *Stetsonia* (pd ≥ 0.980) and from 0.008 to 0.009 J mm^−2^ relative to *Cereus* (pd ≥ 0.992) (Supplementary Table S1).

**Figure 5:**
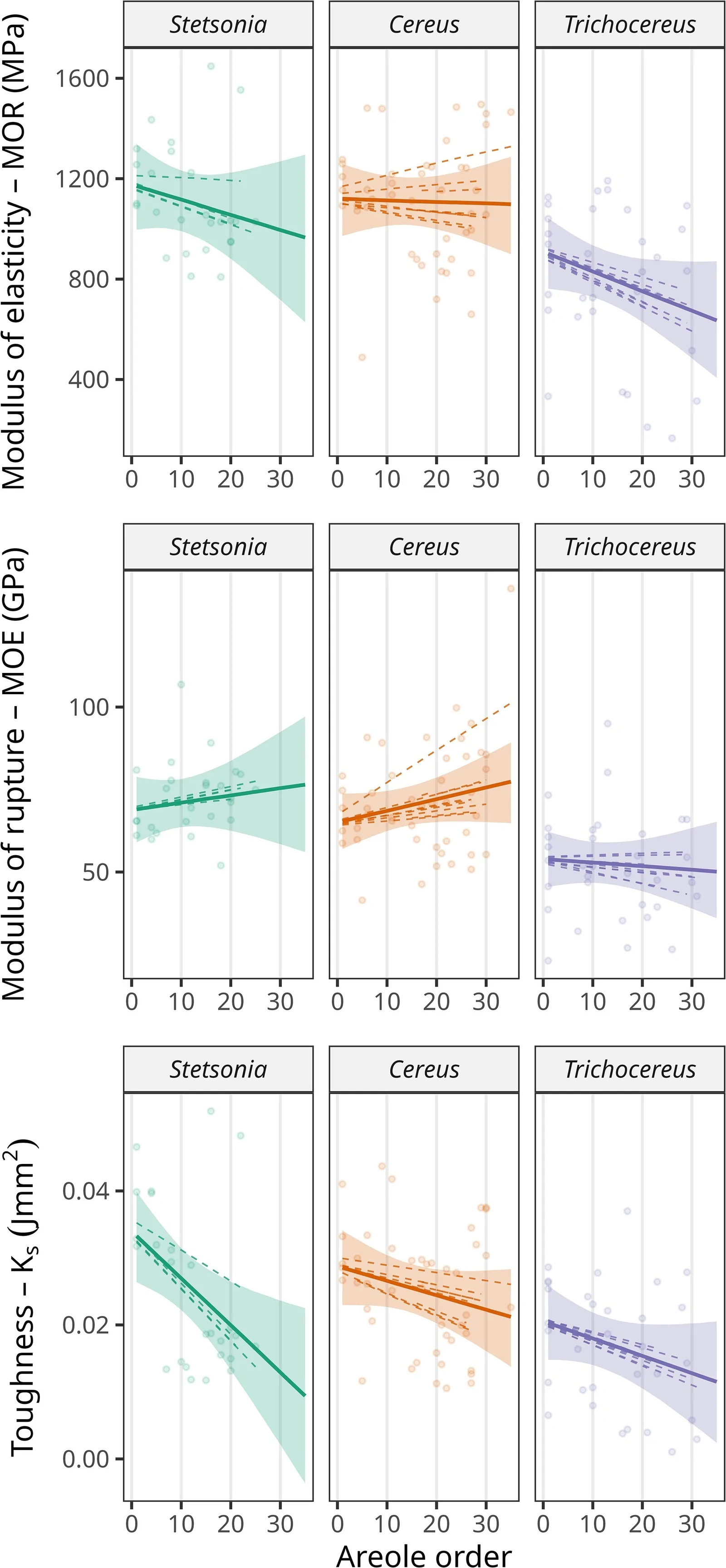
Spine mechanical traits variation along a cactus rib for three columnar cacti species—*Stetsonia coryne*, *Cereus hankeanus* and *Trichocereus terscheckii*. The modulus of elasticity (*MOE*), modulus of rupture (*MOR*), and toughness (*K_S_*) are shown. The mean trends for plant individuals are shown by a dashed line, and the population mean trend for each species is shown by a solid line together with 95% credible interval represented by the shaded area. Points represent measured values. Spine mechanical properties are similar in *S. coryne* and *C. hankeanus*, but lower in *T. terscheckii*. *K_S_* decreases along the stem in all species, but this trend is discernible only in *S. coryne*.

### Areole order

For *Stetsonia* and *Cereus,* spine length increased with areole order, corresponding to increasing distance from the stem apex along a cactus rib (Fig. 4; Table 1 and Table S2). Toughness derived from mechanical traits, however, decreased with increasing distance from stem apex in all three species, although the trend was more pronounced in *Stetsonia*, where the effect size was approximately twice that observed in *Cereus* and *Trichocereus* (Fig. 5; Supplementary Table S2). Anatomical traits were not affected by the distance from the stem apex (Fig. 4; Supplementary Table S2). The proportion of variability explained by the full model (conditional *R*^2^), including species, areole order, and cacti individual, was lower (about half) for toughness (*R*^2^_cond_ = 0.34), modulus of rupture (*R*^2^_cond_ = 0.41), and modulus of elasticity (*R*^2^_cond_ = 0.44) than for spine length (*R*^2^_cond_ = 0.61) (Table 1). In contrast, the variability explained by the fixed effects alone (marginal *R*^2^; species and areole order) was similar across traits (0.24–0.32) (Table 1). This suggests that variability among individual plants accounted for a smaller proportion of total variability in mechanical traits than in spine length (Table 1, Figs. 3 and 5). Nevertheless, considerable within-species variability was observed across all measured traits, with spine length showing the greatest variation, followed by cell wall thickness, fibre maximum and minimum diameters, fibre roundness, modulus of rupture, toughness, and modulus of elasticity (Figs. 3–5; Supplementary Table S3).

## Discussion

### Species comparison

Among the traits measured, spine length showed the greatest variation within the sampled individuals. The longest spines were 5 to 10 times longer than the shortest ones, depending on the species. Variability among plants within species was also highest for spine lengths, followed by cell wall thickness, fibre maximum and minimum diameters, fibre roundness, modulus of rupture, toughness, and modulus of elasticity in descending order. Although the mean spine length of *Stetsonia* discernibly differed from other columnar cacti, the ranges overlapped considerably (Fig. 3). It should also be noted that only the largest spines per each areole were collected in this study, suggesting that the actual intraspecific variation in spine length may be even greater. These findings support previous observations that spine length is highly variable within species (Rundel 1977, Gebauer *et al*. 2016). Because spine length is among the most used morphological characters in cactus taxonomy, such extensive overlap among species and high within-species variation may complicate its use as a standalone diagnostic trait.

The simple anatomical structure of the spine, which consists only of fibres, was confirmed in the two species examined anatomically, *Stetsonia* and *Trichocereus*. Anatomical traits were generally less variable than spine length, as previously observed for *Gymnocalycium kieslingii* subsp. *castaneum* (Gebauer *et al*. 2016). Although species-specific differences in fibre shape, dimensions, and cell wall thickness were detected, substantial overlap remained among species (Fig. 4). Together with the high variability of spine length, these results indicate that no single spine trait is sufficient for reliable species delimitation and identification. Instead, spine diversity appears to reflect the combined effects of species-specific differentiation, developmental processes, and environmental variation, highlighting the importance of considering multiple morphological and anatomical traits simultaneously when conducting functional and comparative studies of cacti. Although the ecological consequences of spine traits were not directly tested in the present study, evidence from other spiny plant systems suggests that variation in physical defence structures can influence herbivore damage. For example, in *Acacia sieberiana*, trees with longer and more numerous thorns experienced lower browsing damage, indicating that differences in defensive structures may translate into ecologically relevant differences in plant–herbivore interactions (Babah Daouda *et al*. 2025). Despite these findings highlighting pronounced within-species variability, it is important to acknowledge limitations of our sampling design. Only 10 individuals per species were analysed, all originating from a single population at one locality. Given the broad geographic ranges and ecological heterogeneity of the studied species, our dataset may not fully capture the total extent of intraspecific variation. Therefore, the observed patterns should be interpreted primarily at the level of local population variability. Nevertheless, even within this limited sampling, substantial variability was observed across most traits, although its magnitude differed among trait groups, being most pronounced for morphological characteristics and comparatively lower, yet still notable, for anatomical and mechanical properties.

While part of this variation reflects biological differences among species and individuals, comparisons of mechanical traits across studies must also consider methodological sources of variation. Mechanical properties of cactus spines vary widely across studies due to differences in methodology, including testing approach, sample preparation, and calculation assumptions. This contributes to the wide range of measured mechanical properties from 1.13 GPa (Schlegel 2009) to 33.6 GPa (Gindl-Altmutter and Keckes 2012) for mean values of modulus of elasticity, and approximately from 180 MPa (Cooper *et al*. 2013) to 835 MPa (Kundanati *et al*. 2022) for mean values of modulus of rupture. The values observed in this study were generally higher than those reported elsewhere (Malainine *et al*. 2003, Schlegel 2009, Gindl-Altmutter and Keckes 2012, Cooper *et al*. 2013, Huang and Guo 2013, Martinez *et al*. 2017, Kundanati *et al*. 2022), which may partly reflect the use of oven-dried samples tested immediately after drying. The relatively high mechanical properties values correspond to Mosco (2009), who suggests that cell wall thickness above 3 µm is characteristic of more rigid spines. The mean cell wall thickness in both *Stetsonia* and *Trichocereus* exceeded this threshold. However, despite having higher mean cell wall thickness, *Trichocereus* exhibited significantly lower mechanical properties than *Stetsonia*. Conversely, mechanical properties did not differ between *Stetsonia* and *Cereus*. These results suggest that cell wall thickness alone cannot fully explain variation in spine mechanical properties.

The modulus of rupture and modulus of elasticity of *Stetsonia* and *Cereus* were close to natural polymers as hemp, flax, or even pure cellulose (Ashby 2005). The high mechanical performance of cactus spines is consistent with their lignocellulosic composition, dominated by cellulose and hemicelluloses (arabinan) with smaller amount of lignin (Malainine *et al*. 2003, Huang and Guo 2013). Variation in the composition and relative abundance of cell-wall polymers may contribute to differences in mechanical properties among species and among spines within individual plants. Moreover, chemical traits may provide valuable taxonomic markers, as lignin-derived compounds have been shown to discriminate cactus taxa at both genus and tribe levels (Reyes-Rivera *et al*. 2020). Integrating, chemical, structural, and mechanical analysis could therefore improve our understanding of spine functional diversity, strengthen species identification, and facilitate the selection of cacti as models for bio-inspired, high-performance materials.

### Areole order

Because spines are produced sequentially at the stem apex and remain preserved along the stem, they form a developmental gradient within an individual plant. Similar developmental gradients are known to contribute to within-individual variation in other plant organs, particularly leaves, where morphological and structural traits often vary systematically with organ age and position (Herrera *et al*. 2015, Sánchez-Bermejo *et al*. 2023). Spine traits may likewise reflect both developmental stage and environmental conditions experienced during their formation. Previous studies have also shown that variation in spine traits can be linked to climatic factors, especially precipitation, and temperature (e.g. Rundel 1977, English *et al*. 2007, Menezes *et al*. 2015). In our study, spine length increased with distance from the apex in *Stetsonia* and *Cereus*. A simple explanation for this pattern could be spine age, as spines located farther from the apex are older. However, spine development in cacti is very rapid, and spines reach their final size within a few weeks (approximately 30–60 days) (English *et al*. 2010, Miravel-Gabriel *et al*. 2024). Thereafter, they do not elongate further and remain attached to the stem for many years. Therefore, the observed increase in spine length with distance from the apex cannot be explained by continued elongation of older spines. One possible explanation is a response to temporal variation in environmental conditions during spine formation. However, climatic data from the studied period do not fully support this interpretation as precipitation in the studied localities increased from 2013 to 2015 (Fig. 2), whereas the trend reported by Menezes *et al*. (2015) would predict the opposite trend. Alternatively, the observed pattern may reflect intrinsic developmental or functional factors, such as trade-offs between protection, shading, and resource allocation (Nobel 1983, Gibson and Nobel 1986, Mauseth 2006, Majure 2007, de la Rosa-Manzano *et al*. 2016). Regarding mechanical traits, only toughness decreased with increasing distance from the apex, with the strongest effect observed in *Stetsonia* and weaker but comparable trends in the other species. Since anatomical traits did not vary with position, the decrease in toughness is most plausibly related to spine ageing and gradual degradation of material properties, as spines located farther from the apex are also older. However, not all mechanical traits exhibited this pattern. In particular, modulus of elasticity did not vary with position, which is consistent with Schlegel (2009) who reported no differences in modulus of elasticity between young spines located closer to the stem apex and old spines located further from the apex. This suggests that ageing may affect specific aspects of mechanical performance (e.g. toughness) without altering others (e.g. stiffness) indicating a differential sensitivity of mechanical traits to ageing processes. At the same time, our data are derived from a single population per species, therefore, the observed trends should be interpreted cautiously. Regardless of the underlying mechanisms responsible for these patterns, the observed variation along the stem highlights the importance of sampling position when comparing spine traits among individuals, populations, or species. Similar considerations have emerged from studies of other plant functional traits, where within-individual variation has been shown to contribute substantially to total trait variability (Westerband *et al*. 2021). Accounting for this source of variation is particularly relevant for taxonomic and ecological studies, where spine traits are increasingly used as sources of biological and environmental information.

## Conclusion

This study demonstrates substantial morphological, anatomical, and mechanical variation in spines among three columnar cacti. Variability was observed not only among species but also within species, and among spines within individual plants, even under a relatively constrained sampling design. This indicates that spine traits reflect both species-specific differentiation and developmental processes operating within individual plants. The overlap of several traits among species highlights the importance of considering morphological, anatomical, and mechanical traits jointly when evaluating spine diversity in cacti. Our results also show that variation along the stem represents an important component of overall spine trait diversity and should be considered in ecological and taxonomic studies. The observed patterns indicate that spine traits may be influenced by developmental position, age, and environmental conditions, although their relative contributions could not be disentangled in the present study. Future research should expand sampling across populations, environmental gradients, and phylogenetic lineages and integrate mechanical measurements with anatomical, chemical, genetic, and environmental data. Such approaches would improve our understanding of spine development and function, clarify the ecological and taxonomic significance of spine traits, and support the use of spine structure as models for the development of bioinspired materials.

## Supplementary Material

plag050_Supplementary_Data

## Data Availability

Raw data and R code are available as Supporting Information.
