# Supplementary material for: Morphological, anatomical, and mechanical variation in cactus spines across species and stem developmental positions
Source: AoB Plants. 2026 Oct 5;18(5):plag050. doi: 10.1093/aobpla/plag050 (PMC13663805; doi:10.1093/aobpla/plag050)
Supplement: Supplementary file 1 [file plag050_supplementary_data.zip › Supplementary Figures.docx]

Supplementary Figures

Fig. S1 Spine cross-section of three columnar cacti growing in Argentina. a) *Stetsonia coryne*; b) *Cereus* [*hankeanus*](http://www.llifle.com/Encyclopedia/CACTI/Family/Cactaceae/6979/Cereus_hankeanus); c) *Trichocereus terscheckii*. All cacti species had simple anatomical structure of spines consist of epidermis and fibers only.

Fig. S2 Fiber maximum and minimum diameters (*d_max_* and *d_min_*), and cell wall thickness (*CW_th_*) were measured for the ten largest fibers in each spine. A representative fiber is highlighted by a dashed black line.

Fig. S3 Three-point bending setup for mechanical destructive tests of spines.
