# Supplementary material for: Morphological, anatomical, and mechanical variation in cactus spines across species and stem developmental positions
Source: AoB Plants. 2026 Oct 5;18(5):plag050. doi: 10.1093/aobpla/plag050 (PMC13663805; doi:10.1093/aobpla/plag050)
Supplement: Supplementary file 1 [file plag050_supplementary_data.zip › Table S1- R3.docx]

Tab. S1 Comparison of spine morphological, anatomical and mechanical traits among three columnar cactus species at the 5^tH^, 15^th^ and 25^th^ areole position from the cacti apex. Spiece include *Stetsonia* (S) and *Trichocereus* (T) and *Cereus* (C). Estimated group means for S are presented with a 95% credible interval (CI). Absolute differences between species pair — S vs. C (ΔSC), S vs. T (ΔST), and C vs. T (ΔCT) — are shown with 95% CI and the probability of direction (pd), defined as the probability that an effect is either positive or negative – whichever is more likely. Estimates with pd > 0.975 are highlighted in bold.

| Trait | | Species effect | | | | | | |
| --- | --- | --- | --- | --- | --- | --- | --- | --- |
|  |  | S (CI) | ΔSC(CI) | ΔSC - pd | ΔST (CI) | ΔST – pd | ΔCT (CI) | ΔCT - pd |
| **Estimates for the 5^th^ areole from cacti apex** | | | | | | | | |
| morpho-logical | spine  length | 61.06 (53.96, 67.87) | **-20.67 (-32.46, -8.80)** | **0.999** | **-21.61 (-33.04, -9.77)** | **0.999** | -0.86 (-12.7, 11.15) | 0.562 |
| Anatomical | *d_max_* | 14.58 (13.62, 15.49) | **-** | **-** | **2.14 (0.67, 3.63)** | **0.997** | - | - |
|  | *d_min_* | 9.47 (8.63, 10.31) | - | - | **2.48 (1.09, 3.89)** | **0.999** | - | - |
|  | *F_r_* | 1.59 (1.50, 1.68) | - | - | **-0.17 (-0.30, -0.04)** | **0.993** | - | - |
|  | *CW_th_* | 4.04 (3.57, 4.49) | - | - | **0.88 (0.17, 1.62)** | **0.990** | - | - |
| mechanical | *MOR* | 1147 (1006, 1279) | -29 (-209, 153) | 0.627 | **-276 (-458, -88)** | **0.997** | **-247 (-412,-75)** | **0.997** |
|  | *MOE* | 69.86 (61.75, 77.85) | -3.14 (-13.85, 7.67) | 0.718 | **-16.51 (-27.11, -5.76)** | **0.998** | **-13.43 (-23.30,-3.43)** | **0.995** |
|  | *K_S_* | 0.030 (0.025, 0.035) | -0.003 (-0.010, 0.004) | 0.780 | **-0.011 (-0.014, -0.004)** | **0.999** | **-0.008 (-0.014,-0.002)** | **0.992** |

| **Estimates for the 15^th^ areole from cacti apex** | | | | | | | | |
| --- | --- | --- | --- | --- | --- | --- | --- | --- |
| morpho-logical | spine  length | 69.08 (62.47, 75.68) | **-22.37 (-33.95, -11.03)** | **1.000** | **-27.89 (-38.85, -16.56)** | **1.000** | -5.49 (-16.74, 5.91) | 0.837 |
| Anatomical | *d_max_* | 14.06 (13.16, 15.01) | - | - | **2.56 (1.09, 3.98)** | **0.999** | - | - |
|  |  |  |  |  |  |  |  |  |
|  | *d_min_* | 9.39 (8.63, 10.27) | - | - | **2.56 (1.22, 3.76)** | **0.999** | - | - |
|  | *F_r_* | 1.53 (1.45, 1.60) | - | - | **-0.12 (-0.22, 0.00)** | **0.977** | - | - |
|  | *CW_th_* | 4.08 (3.71, 4.48) |  |  | **0.83 (0.23, 1.41)** | **0.994** |  |  |
| mechanical | *MOR* | 1086 (9523, 1211) | 24 (-140, 192) | 0.615 | **-295 (-463, -115)** | **0.999** | **-319 (-460, -172)** | **1.000** |
|  | *MOE* | 72.09 (64.11, 79.87) | -1.88 (-11.91, 8.56) | 0.647 | **-19.81 (-30.11, -8.92)** | **0.999** | **-17.95 (-26.57, -9.05)** | **1.000** |
|  | *K_S_* | 0.023 (0.019, 0.028) | 0.002 (-0.004, 0.008) | 0.743 | **-0.007 (-0.013, -0.000)** | **0.980** | **-0.009 (-0.014, -0.004)** | **0.999** |

**Estimates for the 25^th^ areole from cacti apex**

| morpho-logical | spine  length | 77.10 (68.89, 85.61) | **-24.09 (-38.09, -10.42)** | **1.000** | **-34.14 (-47.72, -20.48)** | **1.000** | -10.04 (-23.28, 3.30) | 0.934 |
| --- | --- | --- | --- | --- | --- | --- | --- | --- |
| Anatomical | *d_max_* | 13.53 (11.99, 15.17) | **-** | **-** | **2.98 (0.64, 5.16)** | **0.992** | - | - |
|  | *d_min_* | 9.31 (7.90, 10.89) | - | - | **2.63 (0.37, 4.63)** | **0.986** | - | - |
|  | *F_r_* | 1.47 (1.34, 1.61) | - | - | -0.07 (-0.25, 0.12) | 0.761 | - | - |
|  | *CW_th_* | 4.12 (3.45, 4.82) | - | - | 0.79 (-0.21, 1.72) | 0.945 | - | - |
| mechanical | *MOR* | 1025 (807, 1240) | 79 (-180, 337) | 0.730 | **-313 (-583, -32)** | **0.985** | **-391 (-590, -182)** | **1.000** |
|  | *MOE* | 74.30 (61.15, 87.42) | -0.54 (-16.19, 15.13) | 0.582 | **-23.15 (-39.72, -5.73)** | **0.995** | **-22.61 (-34.72, -9.15)** | **0.999** |
|  | *K_S_* | 0.017 (0.008, 0.025) | 0.007 (-0.003, 0.017) | 0.920 | -0.002 (-0.013, 0.008) | 0.678 | **-0.009 (-0.017, -0.001)** | **0.989** |
