# Supplementary material for: Morphological, anatomical, and mechanical variation in cactus spines across species and stem developmental positions
Source: AoB Plants. 2026 Oct 5;18(5):plag050. doi: 10.1093/aobpla/plag050 (PMC13663805; doi:10.1093/aobpla/plag050)
Supplement: Supplementary file 1 [file plag050_supplementary_data.zip › Table S2 - R3.docx]

Tab. S2 The influence of areole order from the cacti apex on spine morphological, anatomical and mechanical traits of three columnar cacti species: *Stetsonia* (S), *Cereus* (C) and *Trichocereus* (T). Slope S, C and T are presented with a 95% credible interval (CI) and the probability of direction (pd), defined as the probability that an effect either positive or negative – whichever is more likely. Slopes with pd > 0.975 are highlighted in bold. For each trait, results are based on the same statistical models as in Tab. S1 (one model per trait). However, the focus here is on slope estimates for the effect of areole order.

|  | Trait | Areole order effect | | | | | |
| --- | --- | --- | --- | --- | --- | --- | --- |
|  |  | slope S (CI) | slope S – pd | slope C (CI) | slope C – pd | slope T (CI) | slope T - pd |
| morholo-gical | spine length | **0.805 (0.420, 1.197)** | **1.000** | **0.633 (0.293, 0.967)** | **1.000** | 0.174 (-0.189, 0.534) | 0.830 |
| anatomical | *d_max_* | -0.052 (-0.142, 0.041) | 0.874 | - | - | -0.011 (-0.09, 0.068) | 0.609 |
|  | *d_min_* | -0.008 (-0.095, 0.085) | 0.567 | - | - | -0.002 (-0.078, 0.076) | 0.516 |
|  | *F_r_* | -0.006 (-0.015, 0.003) | 0.907 | - | - | -0.001 (-0.008, 0.007) | 0.585 |
|  | *CW_th_* | 0.004 (-0.039, 0.049) | 0.574 | - | - | -0.001 (-0.035, 0.034) | 0.522 |
| mechanical | *MOR* | -6.02 (-18.53, 6.64) | 0.828 | -0.71 (-8.45, 7.15) | 0.572 | -7.86 (-16.57, 0.92) | 0.961 |
|  | *MOE* | 0.219 (-0.517, 0.977) | 0.718 | 0.352 (-0.125, 0.813) | 0.928 | -0.108 (-0.633, 0.426) | 0.654 |
|  | *K_S_* | **-0.0017 (-0.0012, -0.0002)** | **0.997** | -0.0002 (-0.0005, 0.0001) | 0.925 | -0.0003 (-0.0006, 0.0001) | 0.931 |
