# Supplementary material for: Morphological, anatomical, and mechanical variation in cactus spines across species and stem developmental positions
Source: AoB Plants. 2026 Oct 5;18(5):plag050. doi: 10.1093/aobpla/plag050 (PMC13663805; doi:10.1093/aobpla/plag050)
Supplement: Supplementary file 1 [file plag050_supplementary_data.zip › Table S3 - new.docx]

Tab. S3. Standard deviations from 'Tab. 1' are calculated for standardised traits (i.e. the standardised response variable) at the 5^th^ areole. Specifically, the standard deviation for plant and spine levels is reported as a median value and a 95% credible interval. These estimates are useful for comparing variability of traits with each other. It shows that the variability is highest for spine length and lowest for mechanical traits and F_r_ at the plant level. However, it is important to consider the low precision (wide credible interval) of the standard deviation estimates at the plant level for the studied traits (except spine length). Model for F_r_ was fitted with normal distribution here to obtain estimates of standard deviation comparable to other traits.

|  | Trait | Standard deviation – for standardised response; at 5^th^ areole | | | |
| --- | --- | --- | --- | --- | --- |
|  |  | Plant intercept (95% CI) | | Spine (within plant) intercept (95% CI) | |
| morholo-gical | spine length | 0.64 | (0.48, 0.87) | 0.63 | (0.60, 0.66) |
| anatomical | *d_max_* | 0.38 | (0.03, 0.84) | 0.63 | (0.45, 0.84) |
|  | *d_min_* | 0.37 | (0.02, 0.88) | 0.65 | (0.44, 0.87) |
|  | *F_r_* | 0.25 | (0.01, 069) | 0.77 | (0.72, 0.83) |
|  | *CW_th_* | 0.51 | (0.06, 1.00) | 0.56 | (0.36, 0.79) |
| mechanical | *MOR* | 0.22 | (0.01, 0.54) | 0.81 | (0.70, 0.95) |
|  | *MOE* | 0.19 | (0.01, 0.50) | 0.79 | (0.68, 0.93) |
|  | *K_S_* | 0.20 | (0.01, 0.50) | 0.86 | (0.74, 1.01) |
